# Carious Teeth as Carriers of Infection

**Published:** 1912-06-15

**Authors:** T. E. Turner

**Affiliations:** St. Louis


### CARIOUS TEETH AS CARRIERS OF INFECTION.

BY T. E. TURNER, ST. LOUIS.

One of the commissioners of public health of Chicago reported recently, that in the public schools of that city, notwithstanding a rigid process of isolation, disinfection and inspection of scarlatina cases, the re-admission of these patients was almost uniformly followed by fresh outbreak of scarlatina in the school room to which they were returned. This fact attracted so much attention that medical inspectors went over the cases again to see why this occurred, but were inclined to regard the recurrence as merely a matter of coincidence. When cultures were made from cavities in the teeth of these children it was discovered that for a considerable period after the child had returned to school there were germs of scarlatina in these cavities and that fellow scholars were being infected through the common use of drinking cups, lead pencils and books.

Dr. W. A. Evans says: "The major harm is from those decayed teeth being harbingers of bacteria that slowly poison, and as a result of that slow poisoning there is in many instances enlargement of the neighboring glands, and those glands stand as vicarious sacrifices protecting the remainder of the body from the invading poison. And there is nature's route by which the poison finds its way into the body. It is relatively easy to see how teeth decay and how accumulation of filth takes place in those decayed teeth, it is not difficult to see those enlarged glands, but it is far more difficult to understand why the child is pale and anemic. Absorption is taking place from those affected areas and the influence of that absorption is felt not only in the neighboring glands but also in this group of physical conditions that are far removed and the relation of which is difficult to understand. It is not only difficult for the medical man to understand this, but it is far more difficult for the family to understand it."

In regard to scarlet fever he says: "We turned loose lots of children from the hospitals when the scaling had stopped, after which other cases of scarlet fever came back to the hospitals from those families, and that experience has been duplicated all over the world. Now we know that a large number of these returns are due to the causative agents found lingering in the follicles of the tonsils, in cavities in the teeth and other obscure recesses in the mouth and nose."

"A while ago we had an epidemic of diphtheria break out in a Chicago school, we could not run it down. We went through that school and found nine children, and apparently well children at that, who had active diphtheria bacilli in their mouths, they were not sick themselves but were capable of inducing sickness in others. These diphtheria bacilli may remain latent in the nose, throat, tonsils and in cavities in the teeth, and that is something that is not fully understood even by practitioners of medicine who do not know dentistry."

The fact cannot be questioned that decayed teeth are carriers of all the well known bacteria which inhabit the mouth.

That decayed teeth causing a disordered masticating mechanism also cause a train of nutritional disorders because of an inability to properly masticate the food is generally recognized, chronic dyspepsia, intestinal indigestion, and the auto-intoxication caused by absorption of bacterial waste products developed in the fermenting masses of imperfectly masticated and infected food, are examples of pathological conditions caused by inability to properly masticate. Bad teeth mean poor digestion, poor digestion means poor assimilation, poor assimilation means poor health, poor health means poor progress in school studies, a weakened mental efficiency.

In Judge Caldwell's juvenile court in Cincinnati, no child is placed on trial until the judge is satisfied that he is in a normal state of body and mind. The court has no physician, but a number of specialists of high standing volunteer their services, it often happens that a child's delinquency is traceable directly to the state of his health, the defect cured the delinquency departs. This is one of the great discoveries of the juvenile court. Watching a stream of children drifting through a juvenile court you become thoroughly convinced that crime and disease are well-nigh inseparable. No words could frame an indictment of society so terrible as these mute witnesses.

The juvenile court of Chicago has made a closer study of this phase of the delinquent child problem than any other court. Dr. Jas. A. Britton, the dentition home physician, examined 3,646 children during 1909, 2,367, or 65 percent were found to have bad teeth. Dr. Britton says: "We have every reason to believe that an aching tooth is frequently the first cause of irregularity in school attendance, and everyone knows that this irregularity in school attendance is one of the first steps towards the juvenile court."

Turning to the economic side of the question, Dr. Luther H. Gulick, formerly physical director of the New York public schools, now director of the department of child hygiene in the Russell Sage foundation, says: "Investigation has shown that it takes children with defective teeth at least six months longer to complete the eight common school grades than those without defective teeth, that costs the city much money." Dr. Gulick further says, the handicap for a child with decayed teeth is 9 per cent, and Dr. J. N. Hurty, secretary of the Indiana state board of health, says the 9 per cent handicap due to decayed teeth could be wholly removed at an expense of one-fifth that incurred in retaining it. If it cost more the conservation of the physical resources of the children of this country demands it.

William R. Woodbury, M. D., of Boston, says: This problem is not altogether one of benevolence, it is in a large measure a problem of economics. Health is needed for great industrial production, every case of disease involves an immediate economic loss to the community in which such events occur. There is coming a great change in the practice of medicine, surgery and dentistry, and a much greater proportion of the attention of these professions is hereafter to be devoted to prevention rather than cure.

The school clinics in Germany have been in operation a sufficient length of time to demonstrate:

(1) That the time expended in putting the teeth in order was far less than the time formerly lost from toothache and diability caused by diseased teeth.

(2) That the cost of keeping the teeth in order was more than compensated for by better health and a consequent reduction in medical expenses.

(3) That the child became physically stronger, secured a higher average in his studies and was easier to control.

Dr. Kerr, medical officer of health for education to the London county council, after a visit to the Cambridge Dental Institute, says the connection between children's teeth and tuberculosis may not at first sight be quite obvious, and explains that bad teeth mean faulty nutrition, digestive disorders and a consequent weakening of the system, which is therefore less able to resist the attacks of the tubercle bacilli. Consumptives in hospitals, says Dr. Kerr, almost invariably have exceptionally bad teeth, a fact which he has noticed for many years. In establishing the "Infantry" as the Cambridge Dental Institute is called, the founders were building better than they knew. This connection between consumption and bad teeth will no doubt do much to popularize the movement in favor of school dental clinics. It is better to prevent than cure. One of the most important points to which attention has been directed at the tuberculosis exhibition at Cambridge is the fact that people's state of health has a great deal to do with their power of resisting the attacks of the tubercle bacillus. This is no new thing, of course. Most people realize that a good bodily condition is a great protection against all kinds of disease, but a very important aspect of the case has not received the prominence it deserves, one that is bound to have far reaching results and may go a long way towards realizing the hope that tuberculosis may be stamped out in the course of a generation or two. It is the saving and preservation in good condition of the teeth of the rising generation. Mr. Gant, the dentist in charge of the institute, in answer to an inquiry stated that the cost per child, provided they were taken in hand in good time, before the teeth became badly decayed, was 2s, 6d per annum.

Indisputable evidence is furnished in this country by the Marion school class of Cleveland, which was exhibited at the recent meeting of the N. D. A. This class numbered 27 children, showing the worst oral condition out of 846 pupils as determined by a dental inspection. Miss O'Neill the principal says: We had children of good mentality represented and those of the poorest. In fact, one of the little girls had been pronounced mentally defective by our medical inspector. We had well-meaning, tractable pupils and incorrigibles among the group, three truants among the number were on the verge of being called into the juvenile court, and one boy who had repeatedly given trouble in the school room and playground had his papers made out for his transference to our boys' school, where special attention is given to such pupils. The teeth of these children were put in perfect condition and their mouths kept in a hygienic condition for 14 months, six psychological examinations were given, two before any work was done, two while the teeth were being treated and two after all work had been finished. The examinations were to test memory, spontaneous association, addition, association by opposites and quickness and accuracy of perception. They were conducted by the school authorities and were prepared by Dr. Wallin, a psychological expert. The general average increase was 99.8 percent, one girl making 444 percent. Miss O'Neill says not only was this mental gain made, but there was a physical improvement very marked in every case, and spirit of self-respect was engendered that corrected disobedience, truancy and incorrigibility. Two girls who before had sallow complexions and pimpled faces finished up with clear skins and rosy cheeks, a girl who suffered much from flatulency and sick headache, was entirely relived, a case of kidney trouble was almost entirely cured, a boy was immune from contagion of scarlet fever, though living in a family and helping to nurse six younger members who had contracted the disease. Undoubted proof was established that by keeping the teeth in perfect condition, living up to the laws of oral hygiene, these 27 children doubled their mental ability, gained in power and endurance and bodily strength, showed marked improvement in personal appearance and habits. A physical, mental, moral gain in the child produces an economic and financial gain to the community. Can anyone question that a practical working knowledge of oral hygiene is worth while?—*Dental Review.*

